# Risk assessment of subclinical mastitis in Holstein cows in Jiangsu Province of China

**DOI:** 10.3389/fvets.2026.1863556

**Published:** 2026-07-15

**Authors:** Yan Liang, Zhaozheng Zhang, Shuang Gu, Niel A. Karrow, Jinling Hua, Yongjiang Mao

**Affiliations:** 1 College of Animal Science, Anhui Science and Technology University, Chuzhou, China; 2 College of Animal Science and Technology, Yangzhou University, Yangzhou, China; 3 Department of Animal Biosciences, Center for Genetic Improvement of Livestock, University of Guelph, Guelph, ON, Canada

**Keywords:** Chinese Holstein cow, fixed effects, multi-factor logistic regression, nongenetic factors, subclinical mastitis (SCM)

## Abstract

**Introduction:**

Milk somatic cell count (SCC) and somatic cell score (SCS) are good indicators of the occurrence of subclinical mastitis (SCM) in dairy cows.

**Methods:**

In this study, multi-factor ANOVA model was used to analyze the fixed effects of 6 factors, including farm size, management, parity, calving season, lactation stage, and previous SCS (PSCS), on the current month SCS in milk of dairy cows in a training dataset containing 858,546 dairy herd improvement (DHI) records from 12 dairy farms in Jiangsu Province of China. The Logistic regression models of SCS or SCM were established for each factor and and then valiated with a separate dataset containing 27,575 DHI records from 6 other dairy farms.

**Results:**

The results showed that the six factors had significant effects on SCS and SCM incidence in dairy cows (*p* < 0.01). Among them, milk SCS was significantly higher in the farms with fewer than 2,000 cows and fermented bed breeding, and in the cows having more than 5 parities, calving in summer, in late lactation, and when PSCS was 9. The odds ratios (OR) at different levels of each factor were consistent with the actual incidence of SCM. Among them, SCM incidence and OR of cows were lowest when farms had more than 10,000 cows, free stalls, when cows were in first parity, and during the autumn and mid lactation, and when the PSCS was 0. Prediction accuracy and balanced accuracy of risk of SCM during validation were 87.5 and 56.0%. The multivariable logistic regression model demonstrated fair discriminative ability (AUC = 0.771). However, due to its low sensitivity at the default threshold, the model should not be recommended as a standalone farm-level early warning tool without threshold calibration and external validation.

**Conclusion:**

The model may be useful for risk stratification (e.g., identifying high-risk subgroups), but prospective validation is needed before field application.

## Introduction

Mastitis is one of the most common diseases affecting dairy cows, and it is also the most serious disease in terms of economic loss to the dairy industry. Mastitis significantly reduces milk yield and quality, but also leads to changes in milk composition that can significantly affect its nutritional and edible value (1, 2). Mastitis includes clinical mastitis (CM) and subclinical mastitis (SCM); the later of which is characterized by no obvious abnormal changes to the mammary gland and milk, but with detectable changes in physical and chemical properties and higher than acceptable numbers of bacteria in milk. According to a data released by the United States National Mastitis Council in 2018, 44.7% of dairy cows and 17.1%of milking herds have suffered from SCM, which resulted in losses of $184 per cow per year and $2 billion in total in the United States (3). According to Chinese statistics, CM affects about 30% of dairy cows in China, while the incidence of SCM affects up to 70% (4). Approximately 35% of the total number of eliminated or culled cows in China are due to mastitis, with cows in the early lactation and dry stages being the most susceptible to mastitis. The pathogenic bacteria mainly responsible for bovine mastitis include *Staphylococcus aureus*, *Escherichia coli*, *Streptococcus* spp. and Non-aureus *staphylococci* (5).

High milk somatic cell count (SCC; >200,000 cells/mL) or somatic cell score (SCS) is an indirect indicator of SCM in dairy cows (6), and SCC in milk produced by healthy cows is less than 200,000 cells/mL. Therefore, SCC are used as a tool for herd management and as a phenotype for genetic selection. Many factors affect milk SCC, including farm management (7), parity (8), lactation stage (9), and calving season (10).

The logistic regression (LR) model uses the probability of an event, such as disease and death, as the dependent variable and factors affecting the event’s occurrence as independent variables to establish a regression model, and is especially suitable for binomial or multinomial classification data with the dependent variables (11). LR models are widely used in medical fields. In clinical medicine, epidemiology and social statistics among other fields for example, LR is mainly used for differential diagnosis, evaluation of treatment measures and analysis of factors related to an event (12, 13). Guo et al. (14), for example, determined the independent risk factors of ovarian cancer through the establishment and verification of a LR model to provide a basis for clinical decision-making. Akhtarul et al. (15) also used multinomial LR and ordered LR to compare models and identify influencing factors of anemia. In addition, Bertoncelli et al. (16) predicted the health status of cerebral palsy patients with 90% accuracy based on a LR model.

Application of LR model in risk assessment of SCM is rare, and there are certain differences in model parameters between different regions. Therefore, we sought to predict the probability of SCM by constructing the LR model for assessing SCM in Holstein cows. In this study, the incidence of SCM was applied as the dependent variables, and factors, such as farm size, management, parity, calving season, lactation stage and previous SCS (PSCS), were used as independent variables to establish a LR model for analyzing probability of SCM occurrence. The LR model was then validated with a separate dataset to evaluate the accuracy of the model prediction. Thus, this LR model can be used as a tool for assessing the risk of SCM in dairy farms and to reduce probability of mastitis.

## Materials and methods

### Data collection and collation

DHI system is a production record and management system, mainly to record cows’ milk production and composition, collects herd data, and analyzes them to form testing guidance reports regularly. The report information guides the breeding and management of dairy farms. DHI data since 2010 from 15 dairy farms in Jiangsu province of China were collected, including a total of 1,114,367 DHI records from 107,025 dairy cows, representing about 50% of the milking cows in the province (Supplementary Figure S1; Supplementary Table S1). The data included animal ID number, parity, calving date, testing date, test-day milk yield (TDMY), milk fat content (MFC), milk protein content (MPC), SCS or SCC, and other information. Cow records missing SCC were deleted from the original DHI records. Since SCC values are skewed, they are usually transformed into SCS with a near-normal distribution and then analyzed statistically. Therefore, the obtained SCC data were converted into SCS using the internationally accepted conversion formula SCS = log_2_ (SCC/100,000) + 3 (17). The data set represented number of days in milk (DIM) between 6 and 365 days, milk yield between 5 and 80 kg, SCS between 0 and 9, and SCC records of two adjacent lactating months. After screening, a total of 886,121 DHI records from 78,159 dairy cows were obtained (Supplementary Table S2).

### Statistical analysis

#### Linear mixed model

The least-squares method and linear mixed model (LMM) of SPSS Ver26.0 (IBM, Armonk, New York, NY, USA) was used to analyze the associations between milk SCS and farm size, management, parity, calving season, lactation stage and previous SCS (PSCS) (18, 19). All categorical predictors (farm size, management type, calving season, and lactation stage) were dummy-coded with the most representative category as the reference and were entered into the model as nominal variables. Equation is as follows (Equation 1):


$$\begin{array}{l} Y_{\text{ijklmno}}=\mu +{\mathrm{FS}}_{i}+{\mathrm{FM}}_{j}+{\text{Parity}}_{k}+{\mathrm{CS}}_{l}+{\mathrm{LS}}_{m}\\ +{\text{PSCS}}_{n}+u_{f}+u_{c(f)}+e_{\text{ijklmno}} \end{array}$$ (1)

In the above model, Y*_ijklmno_* is the dependent variable (milk SCS); μ is overall mean; FS*_i_*, FM*_j_*, Parity*_k_*, CS*_l_*, LS*_m_*, and PSCS*_n_* are the same fixed effects as described in the original model; u_f_ ~ N(0, σ_f_^2^) is the random intercept for farm, accounting for clustering of cows within the same farm; u_c(f)_ ~ N(0, σ_c_^2^) is the random intercept for cow (nested within farm), accounting for repeated monthly records from the same cow; e*_ijklmno_* ~ N(0, σ_e_^2^) is the residual error. All random effects were assumed to be independent of each other and of the residuals. Significance of fixed effects was tested using F-tests with Satterthwaite approximation for denominator degrees of freedom.

To account for the hierarchical structure of the data, linear mixed models (LMMs) were fitted with random intercepts for cow ID (to account for repeated monthly records) and farm ID (to account for clustering of cows within farms). Variance components for random effects were estimated using restricted maximum likelihood (REML).

The dairy farms and data information contained in the specific training and test datasets are shown in Supplementary Tables S1, S3.

### Logistic regression model construction

Odds ratio (OR) mainly refers to the ratio of factor A and factor B in SCM divided by the ratio of factor A and factor B in non-SCM, reflecting the correlation strength between the probability of SCM and different factors (20). If OR >1, it means factor A is a risk factor of SCM; if OR <1, it means factor A is a protective factor of SCM.

Based on the results of univariate analysis of DHI data from 12 farms in Jiangsu Province, a statistically significant index (the risk factors of SCM in dairy cows) was determined. The above indexes were substituted into the LR model as selected variables, and LR analysis was conducted by using SPSS ver26.0 software. The equations is as follows (Equation 2):


$$\begin{array}{l} \text{Logist}(P)=\mathrm{Ln}(\frac{p}{1-p})=\beta 0+\beta 1X1+\beta 2X2\\ +\ldots +\beta 6X6+\sigma +e \end{array}$$ (2)

Among them, p/(1–p) is usually used to describe the statistical index of disease occurrence intensity, called Odds; Odds = 1, when the probability P of disease occurrence is equal to the probability 1-P of non-occurrence (i.e., both are 0.5), otherwise, Odds are greater or less than 1. In this study, a SCC greater than 200,000/mL was used as the criterion for the occurrence of SCM. According to Odds, an OR value can be calculated to estimate the influence of this factor on the disease. Specifically, Model 2 is the Logistic risk prediction formula for SCM, where P(y = 0) and P(y = 1) are the probability of cows not suffering from mastitis and cows developing SCM in this month, respectively. P_1_ corresponds to the probability of the occurrence of SCM in cows in the next month. β_0_ is the constant term (the natural logarithm of the ratio of an individual’s probability of developing an illness to the probability of not developing an illness), β_1_ to β_n_ are the coefficients of each variable, respectively (it’s the change in Logit P when you change the independent variable by one unit), X_1_ to X_6_ are the risk factor of SCM, including farm size, management, parity, calving season, lactation stage and PSCS. *σ* is the random-effects parameter for individual cow. e is the random error.

### Logistic regression model validation

The dataset used for the LR model validation included 27,575 DHI data from 9,128 cows on 6 dairy farms (Supplementary Table S3). The model used PSCS along with farm-level and cow-level characteristics to predict SCM status (SCC > 200,000 cells/mL) in the current test month. The LR model was used to analyze the measured indexes in the DHI data. According to the formula below, the Logit(*P*) value can be obtained, and the *p* value can be calculated. The formula is as follows:


$$P=\frac{1}{1+\mathrm{EXP}(-\text{Logit}(P))}$$

*p* > 0.5 was judged to be the predicted occurrence of SCM in the next lactating month. In Judgment Result Type, “0” indicates no SCM, and “1” indicates suffered from SCM, and the consistency between the monthly prediction and the actual situation of the last month’s SCM was compared to verify the accuracy of the model prediction results. If we take “1” and “0” as positive and negative respectively, there are 4 results of the actual classification, as shown below:

Sensitivity, true positive rate, which denotes the proportion of subjects correctly given a positive assignment out of all subjects who are actually positive for the outcome, indicates how well a test can classify subjects who truly have the outcome of interest. In this study, sensitivity specifically refers to the probability that cows with actual SCM are correctly predicted to have SCM. Specificity, true negative rate, which denotes the proportion of subjects correctly given a negative assignment out of all subjects who are actually negative for the outcome, indicates how well a test can classify subjects who truly do not have the outcome of interest (21). In this study, specificity refers to the probability that actual healthy cows are also predicted to be healthy.

TN indicates that the predicted and actual results are consistent and both are healthy. FN indicates that the model predicts cows are healthy, but in reality, cows suffer from SCM, and the results are inconsistent. FP indicates that the model predicts cows will suffer from SCM, but in reality, cows are healthy and the predicted results are inconsistent with the actual results. TP indicates that the predicted and actual results are consistent and both suffer from SCM (Table 1).

When the judgment result type is TN and TP, the model is accurate. The equations are as follows:


$$\begin{array}{l} \text{Accuracy}\;(\%)=\frac{\text{Accurate prediction record}}{\text{Total record}}\\ =\frac{\mathrm{TN}+\mathrm{TP}}{\mathrm{TP}+\mathrm{FN}+\mathrm{FP}+\mathrm{TN}}\times 100\% \end{array}$$

$$\text{Sensitivity}\;(\%)=\frac{\mathrm{TP}}{\mathrm{TP}+\mathrm{FN}}\times 100\%$$

$$\text{Specificity}\;(\%)=\frac{\mathrm{TN}}{\mathrm{FP}+\mathrm{TN}}\times 100\%$$

$$\text{Balanced accuracy}\;(\%)=\frac{1}{2}(\text{Sensitivity}+\text{Specificity})$$

The area under the receiver operating characteristic curve (AUC) was calculated to assess the model’s ability to distinguish between SCM-positive and SCM-negative cows. An AUC of 0.5 indicates random discrimination, while an AUC of 1.0 indicates perfect discrimination.

**Table 1:** Explanation of the actual classification of SCM in cows in the validation set data of 6 farms.

Category		Prediction		Total
		1	0	
Actuality	1	True Positive (TP)	False Negative (FN)	Actual Positive (TP + FN)
	0	False Positive (FP)	True Negative (TN)	Actual Negative (FP + TN)
Total		Predicted Positive (TP + FP)	Predicted Negative (FN + TN)	TP + FN + FP + TN

## Results

### Effects of different factors on milk SCS

As shown in Table 2, the farm size, management, parity, calving season, lactation stage, and PSCS have significant effects on milk SCS (*p* < 0.01). Milk SCS from farms with fewer than 2,000 cows was significantly higher than other farms (*p* < 0.05), and milk SCS from farms with more than 10,000 cows was significantly lower than other farms (*p* < 0.05). Overall, milk SCS decreased as the farm size increased. At the same time, we found that milk SCS from cows with free-stall was the lowest, whereas milk SCS from cows maintained on fermentation beds was the highest, with significant differences between the three management practises (*p* < 0.05). In terms of parities, we found that milk SCS gradually increased with the increase of parity, and there was a significant difference among different parities (*p* < 0.05). For calving season, milk SCS of cows calving in summer and autumn was significantly higher than that of cows calving in other seasons (*p* < 0.05). Also, milk SCS during early and middle lactation was significantly lower than during late and end of lactation (*p* < 0.05). In addition, with the increase of PSCS, milk SCS increased significantly (*p* < 0.05).

**Table 2:** Levels (estimated marginal means ± SE) of somatic cell score (SCS) in milk of cows in Jiangsu province under different fixed effects (farm size, management, parity, calving season, lactation stage and, previous SCS) and corresponding *F* and *p* values.

Factor	Level	DHI record number	SCS	*F* value	*P* value
Farm size	<1,001 cows	20,113	3.02 ± 0.06^a^	4237.869^**^	<0.01
	1,001–2,000 cows	56,705	3.03 ± 0.11^a^		
	2,001-5,000 cows	102,164	2.89 ± 0.09^b^		
	5,001–10,000 cows	92,136	2.65 ± 0.13^c^		
	>10,000 cows	604,682	2.33 ± 0.03^d^		
Management	Free stall	849,993	2.46 ± 1.56^c^	2387.805^**^	<0.01
	Fermentation bed	8,428	3.34 ± 1.69^a^		
	Tie stall	17,379	3.21 ± 1.56^b^		
Parity	1	370,262	2.23 ± 1.88^e^	6214.480^**^	<0.01
	2	253,470	2.45 ± 1.64^d^		
	3	138,682	2.73 ± 1.25^c^		
	4	67,590	3.02 ± 0.92^b^		
	≥5	45,671	3.27 ± 1.31^a^		
Calving season	Spring	92,028	2.46 ± 1.43^b^	666.649^**^	<0.01
	Summer	117,691	2.65 ± 1.54^a^		
	Autumn	426,126	2.63 ± 1.70^a^		
	Winter	239,955	2.44 ± 1.92^b^		
Lactation stage	Early lactation(0–100 d)	241,126	2.31 ± 0.00^c^	4821.386^**^	<0.01
	Mid lactation(101–200 d)	246,941	2.30 ± 0.00^c^		
	Late lactation(201–305 d)	244,745	2.57 ± 0.00^b^		
	The end of lactation(>305 d)	142,988	2.95 ± 0.01^a^		
PSCS	0	222,686	1.79 ± 1.50^j^	19641.680^**^	<0.01
	1	145,772	1.93 ± 1.63^i^		
	2	134,746	2.44 ± 1.63^h^		
	3	93,867	2.99 ± 1.72^g^		
	4	62,590	3.45 ± 1.68^f^		
	5	35,415	3.85 ± 1.41^e^		
	6	20,935	4.24 ± 2.26^d^		
	7	11,773	4.57 ± 2.13^c^		
	8	5,994	4.88 ± 2.02^b^		
	9	2,860	5.10 ± 2.25^a^		

PSCS: previous SCS. *P < 0.05; **p < 0.01; a~i differences in the same column are significant at *p* < 0.05. The same is below.

### Incidence and OR value of SCM risk factors

As shown in Table 3, we found that incidence of SCM and OR was different for cows with different risk factors. SCM incidence was the highest (15.8%) among farms with 1,001–2,000 cows and the lowest (6.4%) among farms with more than 10,000 cows. The OR of a farm size with 1,001–2,000 cows was 1.2 times higher than that of a farm with less than 1,001 cows, and the OR of a farm size with more than 10,000 was 0.5 times higher than that of a farm with less than 1,001, indicating a relatively low probability of SCM.

**Table 3:** Multi-factor (farm size, management, parity, calving season, lactation stage, and previous SCS) logistic regression analysis of subclinical mastitis (defined as SCC > 200,000 cells/mL) regressed against the nongenetic risk factors of 886,121 DHI records from 78,159 dairy cows in 12 farms expressed as odds ratio (OR) and 95% confidence interval (95% CI).

Factor	Level	Incidence %	OR^a^	95% CI
Farm size	<1,001 cows	13.2	1.0	
	1,001–2,000 cows	15.8	1.2^*^	1.173~1.301
	2,001–5,000 cows	12.7	1.0	0.909~1.004
	5,001–10,000 cows	6.5	0.455^**^	0.431~0.481
	>10,000 cows	6.4	0.453^**^	0.432~0.474
Management	Free stall	7.5	1.0	
	Fermentation bed	14.5	2.1^**^	1.954~2.230
	Tie stall	21.8	3.4^**^	3.299~3.575
Parity	1	4.7	1.0	
	2	7.7	1.7^**^	1.661~1.740
	3	10.5	2.4^**^	2.349~2.471
	4	14.1	3.4^**^	3.273~3.469
	≥5	17.7	4.4^**^	4.273~4.552
Calving season	Spring	9.6	1.0	
	Summer	7.9	0.8^**^	0.775~0.829
	Autumn	7.1	0.7^**^	0.697~0.736
	Winter	8.6	0.9^**^	0.852~0.902
Lactation stage	Early lactation (0–100 d)	7.3	1.0	
	Mid lactation (101–200 d)	7.3	1.0	0.973~1.021
	Late lactation (201–305 d)	8.0	1.1*	1.076~1.128
	The end of lactation (>305 d)	10.7	1.5**	1.460~1.555
Previous SCS	0	2.7	1.0	
	1	4.2	1.6^**^	1.514~1.628
	2	5.4	2.1^**^	1.995~2.140
	3	7.8	3.1^**^	2.956~3.171
	4	12.4	5.1^**^	4.946~5.306
	5	21.7	10.0^**^	9.623~10.347
	6	33.6	18.2^**^	17.517~18.930
	7	41.2	25.2^**^	24.112~26.395
	8	45.9	30.6^**^	28.911~32.44
	9	48.7	34.2^**^	31.593~36.989

a Represents the change in SCM incidence for each additional unit level +/− 95 confidence limits (Cl) with reference to the level 1 of each factor. OR > 1 represents an increased incidence compared with level 1, OR < 1 represents a reduction in incidence compared to level 1. *P < 0.05; **p < 0.01.

Farm management also affected risk of SCM. Fermentative bed and tie stall management had higher incidence of SCM and OR than free stall management. Among them, the risk of SCM was the highest (OR = 3.4, incidence = 21.8%) in tie stall compared with free stall, followed by that in the fermentative bed compared with free stall (OR = 2.1, incidence = 14.5%).

With different parities, the incidence and OR of SCM varied and increased as parity increased. The incidence of SCM was the highest in parity > 5 (17.7%) and the lowest in first parity (4.7%), with the OR value of parity > 5 being 4.4 times that of first parity.

The incidence and OR of SCM was also affected by different calving seasons. Among them, the incidence of SCM was the lowest in autumn-calving cows (7.1%), and the highest in spring-calving cows (9.6%). The OR of calving season in summer, autumn and winter were 0.8, 0.7 and 0.9 times those in spring, respectively.

The incidence of SCM and OR also differed depending on lactation stages. SCM incidence was the highest in late lactation cows (10.7%), while it was the lowest in mid-lactation cows (7.3%). The OR of late lactation was 1.3 times that of early lactation, and the OR of late lactation was 1.2 times that of early lactation.

Lastly, SCM incidence and OR was affected by PSCS. The SCM incidence and OR increased with the increase of PSCS. Cows with PSCS equal to 9 had the highest SCM incidence (48.7%), and cows with PSCS equal to 0 had the lowest SCM incidence (2.7%). The OR of PSCS equal to 9 was 34.2 times higher than that of PSCS equal to 0.

### Logistic regression model risk validation

According to the model 2, the Logit(*P*) equation can be obtained from the parameters in Table 4. Among them, all 6 factors of the constant term, farm size, management, parity, calving season, lactation stage, and PSCS reached the highly significant *p* value, and the equation is as follows:


$$\begin{array}{l} \text{Logist}(P)=-6.848-0.201\times X_{1}+0.411\times X_{2}\\ +0.293\times X_{3}–0.045\times X_{4}+0.101\times X_{5}+0.451\times X_{6} \end{array}$$

**Table 4:** The parameters of multi-factor (farm size, management, parity, calving season, lactation stage, and previous SCS) logistic regression model in subclinical mastitis (defined as SCC > 200,000 cells/mL) expressed as coefficients (β), Wald test (χ^2^), *P* value, odds ratio (Exp (B)), and 95% confidence interval (95% CI).

Factor	β^a^	SE.	Wald χ^2^ ^b^	*P* value	EXP(B)^c^	95%CI for EXP(B)
Constant term	−6.848	0.711	92.863	0.000	0.001	—
Farm size(X_1_)	−0.201	0.074	7.312	0.007	0.818	0.707~0.946
Management(X_2_)	0.411	0.068	36.971	0.000	1.508	1.321~1.722
Parity(X_3_)	0.293	0.004	6,041.725	0.000	1.341	1.331~1.351
Calving season(X_4_)	−0.045	0.005	78.306	0.000	0.956	0.947~0.966
Lactation stage(X_5_)	0.101	0.005	395.444	0.000	1.106	1.095~1.117
PSCS(X_6_)	0.451	0.002	37,834.830	0.000	1.570	1.563~1.578

a β are the coefficients of each variable, respectively (it’s the change in LogitP when you change the independent variable by one unit).

b Wald test, in which the estimated value of each parameter is compared with 0 and its standard error is used as a reference.

c Exp (B) is the odds ratio, or relative risk. Let Y = 1 infect SCM and Y = 0 be the control. If the exp (B) > 1 indicates the multiple increase of the probability of disease for each unit increase of the independent variable. If the exp (B) < 1, it indicates that this factor is a protective factor, and the percentage reduction in the probability of disease for each unit increase in the independent variable.

As shown in Table 5, the sensitivity and specificity of the model are 13.6 and 98.3%, the accuracy and balanced accuracy are 87.5 and 56.0%. In addition, ranking the probability points of positive prediction, we found that the actual incidence rate of positive prediction under different probability levels were 66.0% (Top 10%), 63.2% (Top 20%), 64.3% (Top 30%), 63.7% (Top 40%), 61.7% (Top 50%), 59.6% (Top 60%), 58.0% (Top 70%), 56.6% (Top 80%), 55.6% (Top 90%) and 54.8% (Top 100%) respectively (Table 6).


$$\text{Accuracy}\;(\%)=\frac{23,626+506}{27,575}\times 100\%=87.5\%$$

$$\text{Sensitivity}\;(\%)=\frac{506}{506+3,206}\times 100\%=13.6\%$$

$$\text{Specificity}\;(\%)=\frac{23,626}{23,626+417}\times 100\%=98.3\%$$

$$\text{Balanced accuracy}\;(\%)=\frac{1}{2}(13.6+98.3)=56.0\%$$

**Table 5:** Judging the actual incidence of subclinical mastitis (defined as SCC > 200,000 cells/mL) of 27,575 DHI records from 6 farms based on logistic regression model and evaluating the model parameters calculated from the training set data.

Category		Prediction record		Total record
		1	0	
Actuality	1	TP (506)	FN (3,026)	Actual Positive (3,523)
	0	FP (417)	TN (23,626)	Actual Negative (24,043)
Total		Predicted Positive (923)	Predicted Negative (26,652)	27,575

**Table 6:** Actual incidence of subclinical mastitis (defined as SCC > 200,000 cells/mL) of different positive predictive levels from 27,575 DHI records from 6 farms based on logistic regression model.

Rank level ^a^	DHI record number ^b^	Actual Positive record ^c^	Positive accuracy
Top 10%	94	62	66.0%
Top 20%	189	119	63.2%
Top 30%	283	182	64.3%
Top 40%	372	237	63.7%
Top 50%	460	284	61.7%
Top 60%	558	333	59.6%
Top 70%	642	373	58.0%
Top 80%	743	421	56.6%
Top 90%	832	463	55.6%
Top 100%	923	506	54.8%

a For records predicted positive, rank them by *p*-values in descending order and divide them into ten levels.

b All DHI records included in the level.

c Actual number of positive records among the predicted positive records.

**Figure 1:**
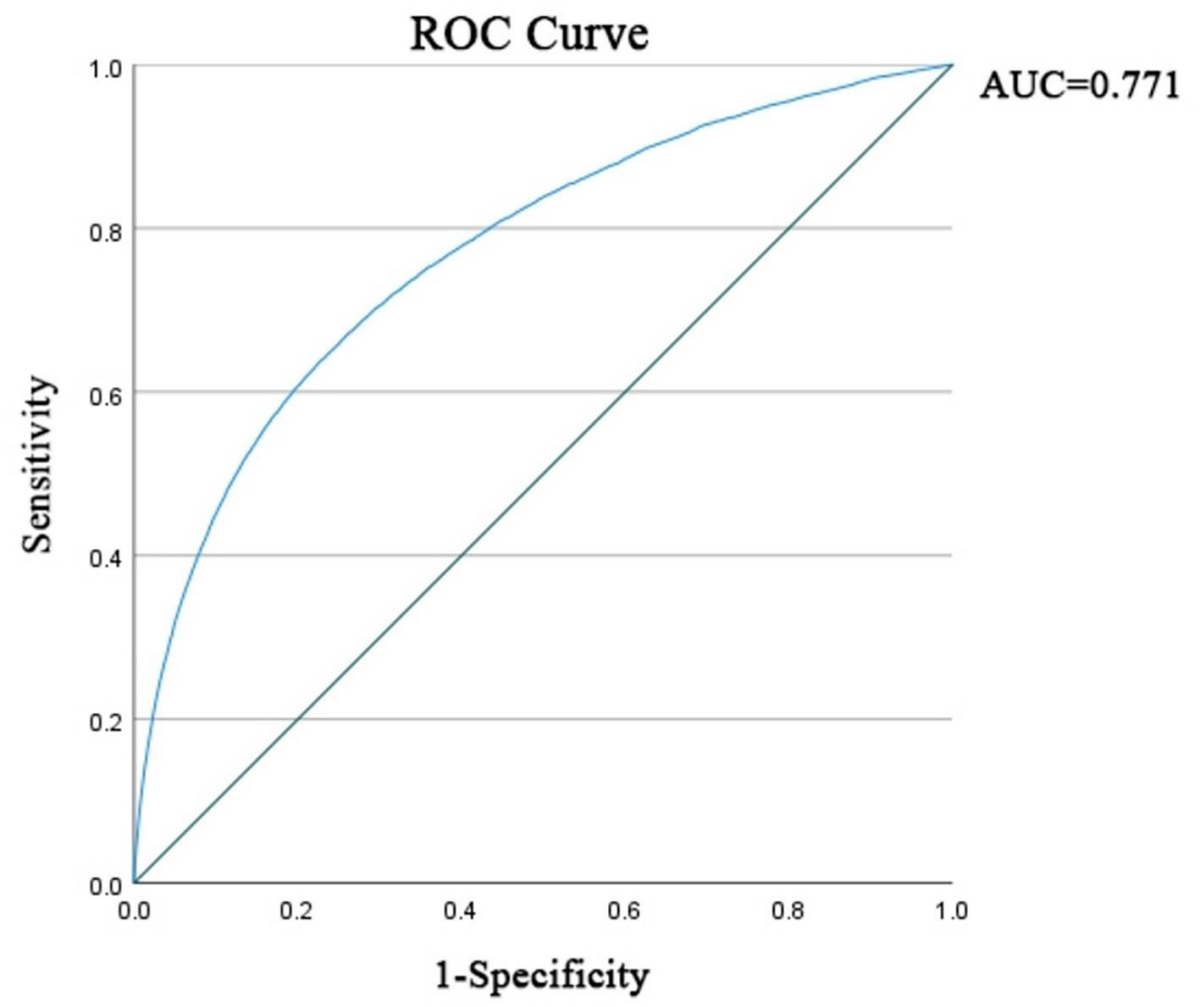
Receiver operating characteristic (ROC) curve for the multivariable logistic regression model predicting subclinical mastitis (SCM).

The ROC curve for the full multivariable logistic regression model is presented in Figure 1. The model achieved an Area Under the Curve (AUC) of 0.771 (95% CI: 0.768–0.774), indicating fair to good discriminative ability (AUC > 0.7). To further evaluate the model’s classification performance across different decision thresholds, we calculated sensitivity and specificity at varying probability cutoffs. As shown in Table 7, the model achieved a maximum Youden’s index of 0.408 at a threshold of approximately 0.080, yielding a sensitivity of 68.8% and a specificity of 72.0%. At the default threshold of 0.5, the model showed high specificity (98.3%) but low sensitivity (13.6%).

**Table 7:** Model performance at different probability thresholds.

Threshold	Sensitivity (%)	Specificity (%)	Youden’s Index
0.50	13.6	98.3	0.119
0.30	26.6	94.1	0.207
0.20	37.7	90.5	0.282
0.15	47.5	86.3	0.338
0.10	61.7	78.2	0.399
0.08	68.8	72.0	0.408
0.07	72.5	67.5	0.324
0.05	81.9	53.3	0.352

Sensitivity and specificity were calculated at each probability threshold using the full logistic regression model. The optimal threshold was determined by maximizing Youden’s index (sensitivity + specificity–1).

## Discussion

As an important index to measure milk quality (SCC) and phenotype for genetic selection to reduce mastitis (SCS), it is important to study factors influencing milk SCC/SCS. A large number of studies have shown that non-genetic factors such as different farms (22, 23), managements (24), parities (25), seasons (25) and lactation stages (9) all have significant effects on milk SCC of dairy cows. Consistently, our study showed that farm size, management, parity, calving season, lactation stage, and PSCS have significant effects on milk SCS in Holstein cows in Jiangsu Province of China. Among them, milk SCS decreased as farm size increased. Gargiulo et al. (26) showed that larger farms with more accurate feeding management technology and cattle management software have a higher ability to prevent and control mastitis. In addition, by grouping cows in different lactation stages and milk yield levels, larger farms with a high degree of mechanization and a strong technical force can reduce the transmission of pathogenic microorganisms and the probability of cow developing intramammary infections that lead to mastitis (27). At the same time, we also found that the milk SCS of free stall cows was the lowest, and the milk SCS of fermentation bedded cows was the highest, with significant differences among different managements. Historical comparison of the three management practices has demonstrated that the bacterial infection rate of free stall cows was lower than that of tie stall cows (28), while the bacterial population in fermentation bed cows was greatly affected by the microbial population in fermenting mat materials (29). Therefore, it may be due to less raking times of fermentation bed, increased moisture content in fermentation bed, and rapid reproduction of harmful bacteria, which is not conducive to udder health, resulting in an increase in SCC in milk in fermented beds. In terms of parities, we found that SCS in milk gradually increased with parities, and there was a significant difference among parities. Jasen et al. (7) also showed that SCC in the milk of dairy cows increased gradually with the increase of parities, and the SCC of cows calving for the first parity was lower than that of cows in higher parities, which was consistent with the results of this study. With the increase of parities, the damage to mammary gland tissue caused by milking increased, and probability of contact and infection with pathogenic microorganisms increased, thus, increasing the chance of intramammary infection and mastitis (30). For the calving season, we found that milk SCS of cows calving in summer and autumn was significantly higher than in the milk of cows calving in other seasons. Riekerink et al. (31) also showed that Holstein cow SCS in summer were higher than those in other seasons. Heat stress reduces dry matter intake and adipose tissue mobilization in cows suffering from summer and autumn climates, and promotes glucose utilization by altering lipid metabolism and hormone signaling pathways, relying on glucose as an energy source for peripheral tissues (32–34), which jeopardizes immune function and increases the risk of infection with pathogenic microorganisms and SCC in milk. Meanwhile, we found that SCS in early and middle lactation cows was significantly lower than in late and end of lactation cows. Relevant studies have shown that, with the increase in lactation period, the number of leukocytes entering the mammary gland from the blood increases, and the function of mammary alveoli is affected (35), causing increased SCC in end-lactation milk. In addition, significant differences in serum nutritional status indicators and physiological status of dairy cows have been observed in cows at different lactation stages, among which the content of vitamin E was the highest in early and middle lactation and then gradually decreased (36); vitamin E levels can reflect the dairy cows’ nutritional status and ability to maintain the balance of oxidants/antioxidants, which is significantly related to milk SCC in dairy cows (37, 38). In addition, with the increase of PSCS, milk SCS increased significantly, which may be related to SCC changes in early lactation leading to SCC changes in late lactation (39).

By calculating the OR of SCM risk factors and the actual incidence, we found that the incidence and OR of SCM were different in cows with different risk factors. Differences in actual incidence and OR between levels of risk factors were also consistent with changes in SCS in milk. Then, we use these factors to construct a LR risk assessment model and obtain the model’s parameters through calculation. We reselected the new DHI data for model validation and found that the predictive accuracy of the SCM risk assessment model was 87.5%, the sensitivity and specificity were 13.6 and 98.3%, respectively. In this study, sensitivity specifically refers to the probability that cows with actual SCM are correctly predicted to have SCM; specificity refers to the probability that actual healthy cows are also predicted to be healthy. Bobbo et al. (40). Compared and analyzed eight different machine learning methods, including logical models, and found that all models exhibited low sensitivity and high specificity. Similarly, Pakrashi et al. (41) compared the sensitivity and specificity of model predictions at different recording frequencies and found that sensitivity and specificity decreased with decreasing recording frequencies. The sensitivity and specificity were 62.1 and 89.1%, respectively, at the same recording frequency as this study. For cases where model sensitivity is low and specificity is high, studies have shown that the sensitivity and specificity of models are inversely proportional (42). More importantly, the difference in the numbers of negative and positive individuals is also a key reason for the difference of sensitivity and specificity (43). Since there are more SCM-negative samples in the data in this study, specificity is more appropriate to evaluate its accuracy. Compared with relevant studies on logical models, although logical models have low sensitivity and high specificity for SCM prediction, logical model is still a valid result to narrow the group of cows that could potentially develop SCM, and the scaleability to differentiate SCM.

### Comparison with recent SCM prediction studies

The performance of our multivariable logistic regression model (AUC = 0.771) is comparable to or slightly lower than recent machine learning-based approaches for subclinical mastitis (SCM) prediction. Han et al. (44) developed a TBESO-optimized BP neural network using monthly DHI data, substantially outperforming conventional regression models. Similarly, ensemble machine learning methods, including gradient boosting and super-learner models, have demonstrated superior performance for SCM classification compared to single logistic regression models (45). A critical challenge shared across SCM prediction studies is class imbalance—the low prevalence of SCM (typically 10–30% at the cow-month level) leads to models with high specificity but low sensitivity when using conventional logistic regression (46). Kashongwe et al. (47) demonstrated that resampling techniques such as SMOTE (Synthetic Minority Oversampling Technique) and SMOTE-ENN can substantially improve sensitivity for mastitis prediction, with some machine learning models achieving sensitivity above 80% after applying appropriate preprocessing. These findings suggest that the low sensitivity of our model (13.6% at default threshold) is not unique but reflects the inherent difficulty of predicting rare events using logistic regression without data-balancing techniques.

In the Chinese context, recent studies have also reported logistic regression-based SCM risk models with comparable performance. A validation study of a cow mastitis logistic regression model (CMLM) across three medium-to-small farms reported average accuracy of 68–69% and specificity of approximately 85%, with similar limitations in sensitivity (46). These studies collectively indicate that while logistic regression remains a practical choice for risk factor identification due to its interpretability, machine learning methods with resampling techniques offer superior predictive performance for SCM detection.

### Class imbalance analysis

Class imbalance refers to the situation in machine learning or data mining where there are significant differences in the number of samples from different categories. In the DHI data used as the training set, there were 109,543 cases of SCM, while there were 749,003 cases of health. The proportion of positive cases was much lower than that of negative cases. Therefore, this also leads to a preference for negative categories during the training process, which affects the model’s ability to recognize positive categories. In common methods for handling class imbalance, such as over/under sampling, threshold tuning, weighted cost function, are not suitable for solving DHI data of cows in practice. Considering the data distribution of positive and negative categories, the probability points of positive prediction are ranked (10% as a rank level, up to 100%), and the actual incidence rate of the cases with the top 10 to 50% were 66.0, 63.2, 64.3, 63.7 and 61.7%, respectively. Upon analyzing the probability points, it was found that the logical model exhibits a good predictive performance for the top 50% of positive prediction probabilities. Therefore, in practical applications, integrating various probability values for segmentation can better enhance the accuracy of positive predictions.

The performance of our multivariable logistic regression model (AUC = 0.771) is comparable to or slightly lower than recent machine learning-based approaches for subclinical mastitis (SCM) prediction. Han et al. (44) developed a TBESO-optimized BP neural network using monthly DHI data, achieving an R^2^ of 0.94 for SCC prediction, substantially outperforming conventional regression models. Similarly, ensemble machine learning methods, including gradient boosting and super-learner models, have demonstrated F1-scores exceeding 0.76 for SCM classification, outperforming single logistic regression models (45).

### Limitations

Several limitations regarding model performance should be noted. First, although the model achieved an AUC of 0.771 (fair to good discrimination), its sensitivity at the default threshold (0.5) was low (13.6%), meaning that the model would miss most SCM cases if used for screening. This is largely attributable to the class imbalance in our dataset (low SCM prevalence), a common challenge in mastitis prediction studies (47). Recent studies have shown that resampling techniques such as SMOTE can substantially improve sensitivity for the minority class, with some machine learning models achieving sensitivity >80% after applying such preprocessing. Second, the optimal probability threshold based on Youden’s index was 0.08, but this threshold requires external validation before practical application. Third, decision curve analysis was not performed; future studies should incorporate this to assess clinical net benefit. Additionally, while our logistic regression model offers interpretability for risk factor identification, recent evidence suggests that ensemble machine learning methods (e.g., gradient boosting, random forest) may achieve superior predictive performance for SCM detection (45). Future research should explore these approaches in conjunction with data-balancing techniques to improve sensitivity while maintaining reasonable specificity.

## Conclusion

In conclusion, risk factors for SCM in Holstein cows were investigated using DHI records and multivariate analysis of variance. The results indicated that disease risk increased with smaller farm size, higher parity, calving during the hot season, later stages of lactation, and larger PSCS. Based on these six factors, a logistic regression model for SCM risk assessment was developed and validated using an independent DHI dataset. The model achieved a prediction accuracy of 87.5% and a balanced accuracy of 56.0%, with a sensitivity of 13.6% and a specificity of 98.3%. The multivariable logistic regression model demonstrated fair discriminative ability (AUC = 0.771). With threshold calibration, the model may support risk stratification; however, external validation is necessary prior to practical application.

## Data Availability

The raw data supporting the conclusions of this article will be made available by the authors, without undue reservation.
