# Supplementary material for: Risk assessment of subclinical mastitis in Holstein cows in Jiangsu Province of China
Source: Front Vet Sci. 2026 Jul 15;13:1863556. doi: 10.3389/fvets.2026.1863556 (PMC13414748; doi:10.3389/fvets.2026.1863556)
Supplement: Supplementary file 1 [file Data_Sheet_1.PDF]

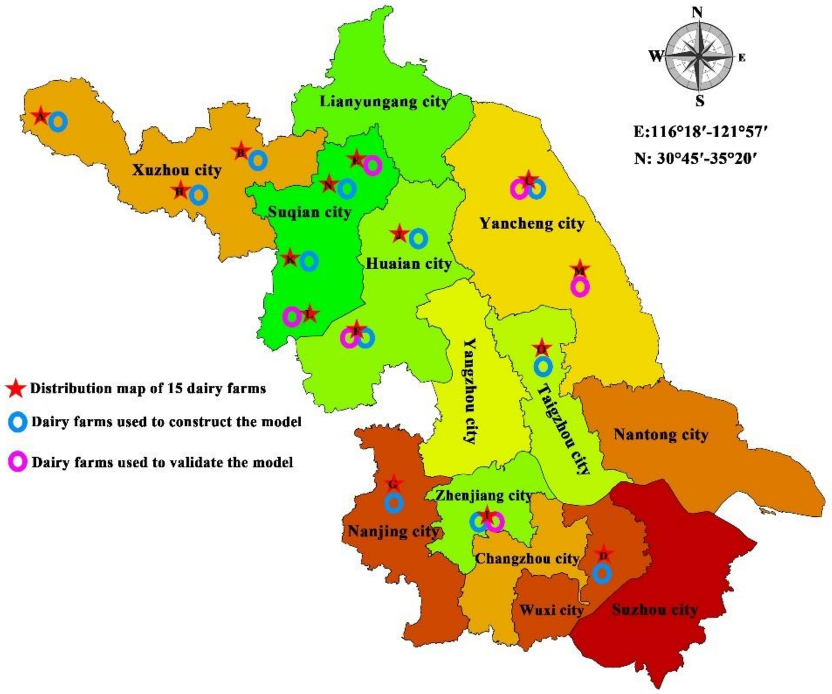

Figure S1. Distribution map of the 15 dairy farms in Jiangsu province used in this study to construct and validate the logistic regression model of subclinical mastitis.

Table S1. Basic information of 12 dairy farms in the training and validation sets

Farm	Farm size (cows)	Management	Average TDMY (kg)[a]	SCC ($\times 10^4$/mL)[b]	FC (%)[c]	PC (%)[d]	MUN (mg/dL)[e]	CI (days)[f]	Pairty (1/2/3/4/5) (%)
A	<1001	Free stall	27.16	18.47	4.15	3.38	12.87	452.06	44.8/33.4/12.5/5.6/3.7
B	<1001	Fermentation bed	29.45	19.13	4.01	3.38	14.21	437.03	49.6/37.3/11.0/2.0/0.1
C	<1001	Tie stall	33.45	28.44	3.63	3.24	13.25	406.93	42.5/28.6/15.0/9.1/4.9
D	1001-2000	Free stall	34.22	15.95	3.91	3.24	11.91	428.47	36.4/32.2/18.3/9.0/4.1
E	1001-2000	Fermentation bed	32.59	18.41	3.55	3.27	13.01	424.73	41.5/24.6/15.2/8.9/9.8
F	1001-2000	Tie stall	37.09	19.61	3.71	3.29	12.87	407.34	33.6/29.5/21.7/10.4/4.8
G	2001-5000	Free stall	32.82	20.71	3.27	3.42	12.72	432.78	41.7/24.2/16.5/8.9/8.7
H	2001-5000	Fermentation bed	33.57	15.08	4.43	3.20	18.13	422.65	32.5/30.6/22.8/9.2/4.9
I	2001-5000	Tie stall	33.82	20.02	3.80	3.33	12.79	395.43	44.1/31.7/15.9/6.3/1.9
J	5001-10000	Free stall	32.14	15.83	3.95	3.40	12.68	409.70	42.1/27.8/16.5/8.4/5.2
K	5001-10000	Fermentation bed	33.40	19.91	3.75	3.34	14.41	419.37	44.0/30.5/16.3/6.5/2.7
L	5001-10000	Tie stall	33.49	18.60	4.00	3.26	13.15	398.46	47.8/25.5/16.1/7.0/3.7
M	>10000	Free stall	35.95	18.62	4.38	3.46	14.34	381.41	47.8/31.2/12.4/5.7/3.0
N	>10000	Fermentation bed	33.58	15.24	3.92	3.33	13.38	452.06	36.7/24.6/14.7/8.8/15.2
O	>10000	Tie stall	27.16	18.47	4.15	3.38	12.87	437.03	34.0/27.9/18.8/9.5/9.7
Mean			29.45	17.13	4.01	3.38	14.21	402.89	40.6/27.4/15.7/7.8/8.4
SE			(0.12)	(12.12)	(0.03)	(0.02)	(0.05)	(9.21)	

[a]TDMY: test-day milk yield.

[b]SCC: somatic cell count.

[c]FC: fat contain.

[d]PC:protein contain.

[e]MUN: urea nitrogen in milk.

[f]CI: calving interval

Table S2. Filtering and processing of number of farms, number of cows and DHI data for training and validation sets [a]

Filter conditions	Number of farms	Number of cows	DHI record number
Datas for recruited	15	101,025	1,114,367
DHI Records without missing	15	93,287	1,037,535
DIM between 6-365	15	88,053	984,755
Milk yield between 5-80 kg	15	83,859	942,987
SCS between 0-9	15	80,846	912,744
Datas for adjacent lactating months were complete	15	78,159	886,121
Data for analysis	15	78,159	886,121
Data for model training [b]	12	75,978	858,546
Data for model validation [c]	6	9,128	27,575

[a] Table S2 number of DHI records enrolled in a retrospective longitudinal study investigating the association between non-genetic risk factors and subclinical mastitis on 15 dairy farms between 2010 and 2021 in Jiangsu Province.

[b] Training dataset：the data used to construct the Logistic regression model came from the total DHI data, including DHI data from 12 dairy farms.

[c] Validation dataset: the data used to validate the accuracy of the constructed Logistic regression model, including DHI data from 3 new dairy farms and new DHI data from 3 of the 12 farms used for the model training.

Table A3. Logistic regression model training and testing datasets

Dataset	Number of farms		Time range	Number of cows	DHI record number
Model training dataset	12[a]		2010/01/15-2019/12/07	75,978	858,546
Model validation dataset	6[b]		2018/01/16-2021/02/24	9,128	27,575
		3[c]	2020/01/18-2021/02/24	6,947	16,299
		3[d]	2018/01/16-2021/02/22	2,181	11,276

[a]: DHI data from 12 dairy farms were used for model construction, and this part of DHI data came from 2010/01/15 to 2019/12/7.

[b]: DHI data from 6 dairy farms were used for model validation, and this part of DHI data came from 2018/01/16 to 2021/02/24. The data of training dataset and testing dataset do not overlap.

[c]: New DHI data from 3 of the 12 dairy farms that were used for model training.

[d]: DHI data from 3 new dairy farms.

Cross-validation

Randomly divide the dataset without replacement into 10 parts, 9 parts for model training, and the remaining part for model performance evaluation. Repeat 10 times to obtain 10 models and performance evaluation results. Calculate the final performance evaluation based on the average value.

Model 1.1	Model 1.2	Model 1.3	Model 1.4	Model 1.5	Model 1.6	Model 1.7	Model 1.8	Model 1.9	Model 1.10	
Train	Train	Train	Train	Train	Train	Train	Train	Train	Valid	Pred 1.1
Train	Train	Train	Train	Train	Train	Train	Train	Valid	Train	Pred 1.2
Train	Train	Train	Train	Train	Train	Train	Valid	Train	Train	Pred 1.3
Train	Train	Train	Train	Train	Train	Valid	Train	Train	Train	Pred 1.4
Train	Train	Train	Train	Train	Valid	Train	Train	Train	Train	Pred 1.5
Train	Train	Train	Train	Valid	Train	Train	Train	Train	Train	Pred 1.6
Train	Train	Train	Valid	Train	Train	Train	Train	Train	Train	Pred 1.7
Train	Train	Valid	Train	Train	Train	Train	Train	Train	Train	Pred 1.8
Train	Valid	Train	Train	Train	Train	Train	Train	Train	Train	Pred 1.9
Valid	Train	Train	Train	Train	Train	Train	Train	Train	Train	Pred 1.10

Figure S2. Cross-validation with 10 replicates to assess the accuracy of the predictive model.

Table S4. All data is randomly divided into 10 datasets for cross validation

Dataset	DHI record number	Number cows
Dataset 1	88,591	8,561
Dataset 2	88,612	8,554
Dataset 3	88,583	8,413
Dataset 4	88,565	8,357
Dataset 5	88,612	8,502
Dataset 6	88,675	8,618
Dataset 7	88,592	8,427
Dataset 8	88,631	8,539
Dataset 9	88,634	8,531
Dataset 10	88,626	8,604
All	886,121	85,106

Table S5. Dataset of 10 repeated cross validation models

Model	DHI record of training dataset	DHI record of validation dataset
Model 1.1	797,495	76,502
Model 1.2	797,487	76,575
Model 1.3	797,490	76,567
Model 1.4	797,529	76,679
Model 1.5	797,446	76,488

Model 1.6	797,509	76,604
Model 1.7	797,556	76,749
Model 1.8	797,538	76,693
Model 1.9	797,509	76,552
Model 1.10	797,530	76,545

Table S6. The parameters of cross-validation Model 1.1 to Model 1.10

Model	Constant term	X_1	X_2	X_3	X_4	X_5	X_6
Model 1.1	-6.197	-0.223	0.404	0.312	-0.046	0.108	0.439
Model 1.2	-6.343	-0.195	0.425	0.268	-0.042	0.112	0.427
Model 1.3	-6.502	-0.183	0.393	0.295	-0.051	0.105	0.441
Model 1.4	-7.102	-0.236	0.396	0.334	-0.045	0.113	0.426
Model 1.5	-6.914	-0.297	0.374	0.317	-0.043	0.097	0.452
Model 1.6	-6.627	-0.254	0.452	0.207	-0.042	0.094	0.407
Model 1.7	-6.769	-0.168	0.427	0.294	-0.048	0.085	0.436
Model 1.8	-7.315	-0.215	0.401	0.275	-0.053	0.097	0.445
Model 1.9	-7.003	-0.227	0.416	0.326	-0.046	0.089	0.409
Model 1.10	-7.572	-0.184	0.454	0.394	-0.039	0.118	0.453
Average value	-6.834	-0.218	0.414	0.302	-0.046	0.102	0.434

$$\text{Logist}(P) = -6.834 - 0.218 \times X_1 + 0.414 \times X_2 + 0.302 \times X_3 - 0.046 \times X_4 + 0.102 \times X_5 + 0.434 \times X_6.$$

Table S7. Judging the actual incidence of SCM (defined as SCC>200,000 cells/mL) based on logistic regression model 1.1 to model 1.10, and use it to evaluate the model parameters calculated from the training set data.

Model	Judgment result type (Records)				Accuracy	Sensitivity	Specificity
	TN	FN	FP	TP			
Model 1.1	75,923	9,704	1,274	1,725	87.6%	15.1%	98.3%
Model 1.2	76,431	9,639	911	1,653	88.1%	14.6%	98.8%
Model 1.3	76,709	9,538	767	1,617	88.4%	14.5%	99.0%
Model 1.4	75,952	9,629	1,279	1,732	87.7%	15.2%	98.3%
Model 1.5	76,521	8,971	1,524	1,659	88.2%	15.6%	98.0%
Model 1.6	74,854	9,345	2,651	1,762	86.5%	15.9%	96.6%
Model 1.7	76,094	9,690	1,122	1,659	87.8%	14.6%	98.5%
Model 1.8	76,134	9,728	918	1,803	88.0%	15.6%	98.8%
Model 1.9	76,752	9,194	977	1,689	88.5%	15.5%	98.7%
Model 1.10	76,569	9,567	711	1,744	88.4%	15.4%	99.1%
Average value	76,194	9,501	1,213	1,704	87.9%	15.2%	98.4%

The random effect variance components are presented in Supplementary Table S8. The variance estimates for cow-level (0.423) and farm-level (0.160) random intercepts were both significantly different from zero ($P < 0.01$), confirming the presence of significant clustering at both levels.

Table S8. Random effect variance components from the linear mixed model

Random effect	Variance	SE	Wald Z
Cow ID (intercept)	0.423	0.004	110.074
Farm ID (intercept)	0.160	0.068	2.343
Residual	2.548	0.004	635.514
